# Polyphenolic compounds in *Glycyrrhiza* spp.: a potential weapon against multidrug-resistant pathogens

**DOI:** 10.3389/fphar.2026.1686787

**Published:** 2026-02-24

**Authors:** Muhammad Rafiq, Zhaoqi Xie, Shahida Hameed, Haijing Xiao, Yiqing Luo, Kai Chen, Jiaxin Yang, Lei Hu, Min Guo, Chunsong Cheng

**Affiliations:** 1 Jiangxi Key Laboratory for Sustainable Utilization of Chinese Materia Medica Resources, Lushan Botanical Garden, Chinese Academy of Sciences, Jiujiang, China; 2 Lushan Xinglin Institute for Medicinal Plants, Jiujiang Xinglin Key Laboratory for Traditional Chinese Medicines, Jiujiang, China; 3 School of Chemistry and Chemical Engineering, Anhui University, Hefei, Anhui, China

**Keywords:** adjuvants, AMR, biofilms, chalcones, flavonoids, glycyrrhiza, MDR bacteria, polyphenols

## Abstract

Antimicrobial resistance (AMR) is a rapidly escalating global health crisis, projected to cause 10 million deaths annually by 2050 without effective intervention. Conventional antibiotics are increasingly compromised by multidrug-resistant (MDR) pathogens that deploy mechanisms such as efflux pumps, membrane impermeability, target modification, and biofilm formation. Polyphenolic compounds from Glycyrrhiza spp (*licorice*) represent a promising, multi-target strategy to counter these threats. This review critically examines the antimicrobial potential of licorice-derived flavonoids, chalcones, coumarins, and glycosides, focusing on their ability to inhibit efflux pumps, disrupt bacterial membranes, prevent biofilm formation, modulate quorum sensing, and synergize with conventional antibiotics. We integrate mechanistic insights with safety, pharmacokinetic, and formulation considerations, highlighting both therapeutic potential and translational challenges. Evidence indicates that licorice polyphenols can restore antibiotic efficacy against MDR bacteria, reduce the likelihood of resistance development, and be incorporated into topical agents or antimicrobial materials. However, limitations in bioavailability, dose-dependent toxicity, and a paucity of clinical trials underscore the need for targeted delivery systems and rigorous *in vivo* validation. By framing licorice polyphenols as risk-mitigating agents, this review positions them as viable candidates for next-generation adjunctive therapies and preventive strategies in the fight against AMR. In particular, their role against high-priority ESKAPEE pathogens and their inherent antioxidant properties further enhance their therapeutic value. This review therefore not only synthesizes current knowledge but also outlines future directions for translating licorice-derived polyphenols into clinical and industrial applications.

## Introduction

Antimicrobial resistance (AMR) has escalated into a global health hazard, with drug-resistant “superbugs” causing over 1.27 million deaths annually and projections of 10 million fatalities per year by 2050 if unchecked (Almutairy, 2024). The World Health Organization now ranks AMR among the top three threats to public health (Salam et al., 2023). Conventional antibiotics are losing efficacy at an alarming rate, as bacteria evolve multi-faceted defense mechanisms (efflux pumps, biofilms, enzymatic drug destruction, target modification, etc.) that render treatments ineffective. This grim outlook has catalyzed the search for novel, risk-mitigating antimicrobials beyond the traditional small-molecule antibiotics. In this context, natural product derivatives–especially plant polyphenols–have emerged as promising leads. Plant secondary metabolites can inhibit bacterial growth and even restore antibiotic potency through mechanisms distinct from classic drugs (Abdallah et al., 2023; Elshafie et al., 2023; Luo et al., 2024). Harnessing such compounds is increasingly seen as an innovative strategy to combat the global hazard of AMR.

**FIGURE 1:**
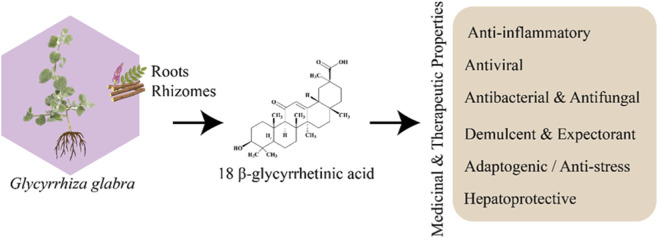
Overview of *Glycyrrhiza spp*. (licorice) medicinal potential. Illustration shows the plant source, representative chemical structure of glycyrrhizin, and major pharmacological activities including anti-inflammatory, antiviral, antibacterial, antifungal, demulcent, expectorant, adaptogenic/anti-stress, and hepatoprotective effects.

Glycyrrhiza has also been studied for its potential to enhance the activity of conventional antibiotics. Research suggests that licorice extracts may act synergistically with antibiotics, enhancing their efficacy against resistant pathogens (Herman and Herman, 2023; Su et al., 2023). The medicinal properties of Glycyrrhiza are attributed to its diverse array of bioactive compounds as shown in Figure 1. These include flavonoids, saponins, and polyphenolic compounds. The primary bioactive constituents of interest are the polyphenolic compounds, which have been shown to possess significant antimicrobial, antioxidant, and anti-inflammatory activities (Hassan et al., 2023; Mączka et al., 2023; Rafiq et al., 2024). Flavonoids such as liquiritin, *glycyrrhizin*, and isoliquiritigenin are among the most studied polyphenolic compounds found in Glycyrrhiza (Wu et al., 2022).

Licorice (*Glycyrrhiza* spp.) – long prized in food and herbal medicine–offers a rich reservoir of bioactive phytochemicals, including triterpene saponins (e.g., glycyrrhizin) and diverse polyphenols (flavonoids, chalcones, coumarins) (Purewal and Singh, 2025; Tan et al., 2025). Notably, *Glycyrrhiza glabra*, *Glycyrrhiza uralensis*, and *Glycyrrhiza inflata* (the principal licorice species) produce a suite of polyphenolic compounds with broad pharmacological activities, antibacterial effects included (Tan et al., 2025; Umarov et al., 2025). Recent research suggests these licorice-derived polyphenols could be potent weapons against multidrug-resistant (MDR) pathogens, acting through unconventional modes such as perturbing bacterial membranes, blocking resistance mechanisms, disrupting biofilms, and synergizing with antibiotics (Cheng et al., 2024; Herman and Herman, 2023). In other words, licorice polyphenols may mitigate the “hazard” of AMR by disarming the pathogen’s defenses and resensitizing it to treatments. This review is framed around that hypothesis, critically examining how *Glycyrrhiza* polyphenols could help curb MDR infections. We focus strictly on polyphenolic compounds and closely related phytochemicals (flavonoids, chalcones, coumarins, and the major saponin glycyrrhizin) from licorice, analyzing their antimicrobial mechanisms, safety profiles, and translational challenges. By scrutinizing primary evidence, we aim to provide a distinctive, analytical perspective on licorice polyphenols as risk-mitigating agents against AMR–a perspective that transcends a simple catalog of activities and instead integrates mechanistic insight with a discussion of practical hurdles on the road from bench to bedside.

Alongside natural product-based strategies, nanomedicine and other innovative therapeutic approaches have gained attention in addressing antimicrobial resistance (Rafiq et al., 2025; Yang et al., 2025). Nanocarrier systems, including liposomes, polymeric nanoparticles, and metal-based nanostructures, can enhance the solubility, stability, and targeted delivery of bioactive compounds such as polyphenols (Karami et al., 2024; Luo et al., 2024). These technologies not only improve bioavailability but also allow co-delivery with antibiotics, thereby potentiating synergistic effects. Incorporating such novel therapeutic modalities provides a forward-looking perspective for harnessing licorice-derived polyphenols in next-generation antimicrobial interventions (Tan et al., 2025; Zhang et al., 2025).

## The AMR challenge and licorice polyphenols as risk mitigating agents

### AMR mechanisms in brief

Bacteria deploy several well-characterized strategies to withstand antibiotics. These include enzymatic degradation or modification of the drug (e.g., β-lactamases that hydrolyze penicillins/cephalosporins) (Su et al., 2023; Wright, 2005), alteration of antibiotic targets (mutations or protective modifications that reduce drug binding) (Darby et al., 2023; Schaenzer and Wright, 2020), and active efflux pumps that expel diverse antibiotics from the cell. In addition, many pathogens form biofilms–structured communities enmeshed in exopolymer matrices–which act as a physical and chemical barrier to antibiotics. Gram-negative bacteria pose extra challenges due to their outer membrane, which restricts drug uptake, and often synergize multiple mechanisms (e.g., porin loss plus efflux and drug-inactivating enzymes) (Saxena et al., 2023). Multidrug-resistant organisms (MDROs) like methicillin-resistant *Staphylococcus aureus* (MRSA), *Enterococcus faecium/faecalis* resistant to vancomycin ((VRE), MDR *Pseudomonas aeruginosa*, *Acinetobacter baumannii*, and certain *Mycobacterium tuberculosis* strains exemplify pathogens that have acquired several of these defenses–making infections extremely difficult to treat (Parmanik et al., 2022). Any prospective anti-MDR agent, therefore, must either evade or counteract these resistance mechanisms.

**FIGURE 2:**
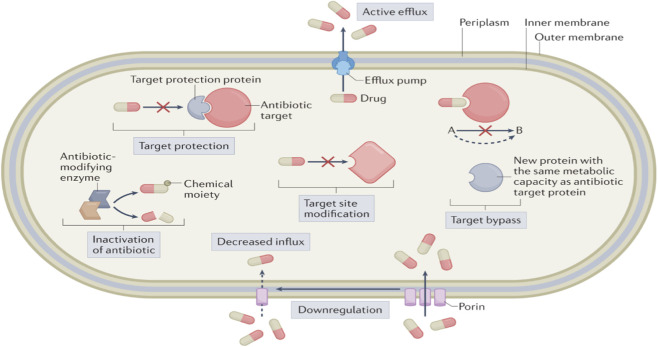
Schematic overview of key bacterial drug resistance mechanisms. The illustration depicts a Gram-negative bacterium (outer membrane in blue) resisting antibiotics (red arrows) through: (1) Efflux pumps actively exporting drugs; (2) Membrane impermeability via reduced porins; (3) Target modification (e.g., ribosomal or enzyme mutations); (4) Biofilm formation creating a protective matrix; (5) Enzymatic degradation (e.g., β-lactamases hydrolyzing β-lactam rings). Gram-positive mechanisms (e.g., cell wall thickening) are also represented for comparison. These strategies highlight the multi-faceted nature of AMR, which licorice polyphenols may counteract by disrupting membranes, inhibiting efflux, or preventing biofilms.

Licorice Polyphenols–A Multi-Target Approach: Polyphenolic compounds from *Glycyrrhiza* show a remarkable concordance with the needs outlined above: they appear to attack bacteria on multiple fronts (Chen et al., 2024). These phytochemicals are generally not substrates for known efflux pumps and often have amphiphilic structures that insert into and destabilize bacterial membranes as shown in Table 1, or otherwise perturb critical cellular processes without being easily nullified by single gene mutations. For instance, the prenylated flavonoids and chalcones in licorice are typically small (<500 Da) lipophilic molecules capable of penetrating cell envelopes and accumulating in bacterial cells (Dang et al., 2024; Zhai et al., 2023). Many polyphenols also carry electrophilic or chelating functional groups that can interfere with enzyme function or cell division in bacteria (Chen et al., 2014; Rodríguez et al., 2023). Importantly, licorice polyphenols have shown activity against clinical MDR isolates *in vitro*–including MRSA, VRE, and drug-resistant *Mycobacterium*–at micromolar concentrations (Rudrapal et al., 2023). By targeting mechanisms like efflux, membranes, and biofilms, these compounds could mitigate the risk posed by superbugs either as stand-alone antimicrobials or as adjuvants that restore antibiotic efficacy as shown in Figure 2. The following sections critically examine key mechanisms by which licorice-derived polyphenols counteract bacterial resistance, along with an evaluation of their potency and limitations.

**TABLE 1:** Antimicrobial resistance (AMR) crisis and potential role of licorice-derived polyphenols in mitigation strategies.

AMR challenge	Mechanism of resistance	Impact on public health	Evidence of licorice polyphenols’ activity	Proposed risk mitigation pathway
Emergence of multidrug-resistant bacteria (MDR)	Efflux pump overexpression, enzymatic degradation of antibiotics	Higher morbidity, mortality, and treatment costs	Licorice flavonoids (e.g., liquiritigenin) inhibit efflux pump activity in Gram-positive and Gram-negative bacteria	Combine polyphenols with existing antibiotics to restore drug sensitivity
Biofilm-associated infections	Biofilm matrix preventing antibiotic penetration	Chronic and recurrent infections, prolonged treatment	Glycyrrhizic acid derivatives disrupt biofilm formation in *Staphylococcus aureus* and *Pseudomonas aeruginosa*	Apply licorice polyphenols in wound dressings and coatings on medical devices
Resistance gene dissemination	Horizontal gene transfer (plasmids, integrons)	Rapid spread of resistance across bacterial populations	Licorice extracts reduce bacterial conjugation efficiency	Use in preventive therapy to limit resistance gene spread
Antibiotic overuse in agriculture	Selection pressure in livestock and aquaculture microbiota	Reservoir of resistant pathogens affecting humans	Antimicrobial activity against *Escherichia coli* and *Salmonella* from livestock isolates	Integrate polyphenols in feed additives to reduce antibiotic dependence
Limited new antibiotic discovery	Decline in novel drug classes	Few treatment options for emerging superbugs	Broad-spectrum inhibitory activity against MDR clinical isolates	Develop polyphenol-based lead compounds as adjunctive antimicrobials

### Efflux pump inhibition by licorice polyphenols

One major resistance mechanism in MDR bacteria is the overexpression of broad-spectrum efflux pumps that expel antibiotics (Huang et al., 2022). In *S. aureus*, for example, the NorA proton-driven pump extrudes fluoroquinolones, reducing intracellular drug levels. Inhibiting efflux pumps is a promising way to rejuvenate older antibiotics, and intriguingly, some *Glycyrrhiza* flavonoids act as natural efflux pump inhibitors (EPIs) (Zhang et al., 2024). A recent study identified glabrene, an isoflavonoid from licorice, as a potent NorA pump inhibitor in *S. aureus* (Ika Irianti et al., 2023; Morante-Carriel et al., 2024). At sub-inhibitory concentrations (6–12 μg/mL), glabrene and a related prenylated isoflavone (neobavaisoflavone) increased intracellular accumulation of ethidium (a NorA substrate) by up to 6-fold and boosted the effectiveness of fluoroquinolone antibiotics by eightfold against a NorA-overexpressing MRSA strain (Hassan et al., 2023; Ika Irianti et al., 2023). Crucially, this synergy disappeared in a NorA knockout mutant, confirming the pump as the target (Chandal et al., 2025). Unlike some generic EPIs (e.g., reserpine), glabrene did not noticeably permeabilize bacterial membranes or exhibit host cell toxicity at active doses (Bombelli et al., 2025), suggesting a specific efflux-targeting action. These findings position glabrene as potential antibiotic adjuvants: by crippling efflux machinery, they can drive previously resistant bacteria to once again accumulate antibacterial drugs to lethal levels.

Licorice also yields other phytochemicals with EPI activity. Licochalcone A, a retrochalcone abundant in *Glycyrrhiza inflata*, has long been noted to enhance intracellular drug retention. While known mostly for its direct antimicrobial effect, licochalcone A and related chalcones carry prenyl side chains that could intercalate in efflux protein pockets or disrupt the proton gradients needed for pump function (Adhikari et al., 2025). Even glycyrrhizin (GLY), the triterpenoid glycoside for which licorice is famed, appears to modulate efflux indirectly. In *Pseudomonas aeruginosa* and other Gram-negatives, glycyrrhizin has been reported to inhibit efflux pump activity and alter cell permeability, thereby reducing bacterial multidrug resistance (Zhang et al., 2024). A 2016 study on *P. aeruginosa* found that licorice extract containing GLY increased intracellular uptake of antibiotics and dyes, consistent with efflux suppression (Chen et al., 2024). The net result was enhanced susceptibility of the bacteria to various antimicrobials. Thus, through both dedicated EPIs like glabrene and more pleiotropic compounds like GLY, *Glycyrrhiza* polyphenols can neutralize one of bacteria’s most formidable defense mechanisms.

From a risk-mitigation standpoint, efflux pump inhibitors are invaluable because they restore the potency of existing antibiotics–effectively rescuing drugs that pathogens had rendered useless. Licorice-derived EPIs have the advantage of being natural products with potentially fewer side effects than synthetic inhibitors, and some (like neobavaisoflavone) have shown lower cytotoxicity and greater potency than reserpine in laboratory comparisons (Ji et al., 2024). However, it must be noted that most evidence so far is *in vitro*. The challenge will be achieving sufficient concentrations of these flavonoids at infection sites *in vivo* to inhibit pumps without off-target effects. Nonetheless, the efflux-targeting activity of licorice polyphenols represents a crucial piece of their anti-MDR repertoire.

## Membrane disruption and structural damage to pathogens

Many licorice polyphenols also exhibit a direct bactericidal mechanism that is physiologically hard for bacteria to resist: they attack the integrity of the cell membrane and cell wall. This mode of action is significant because membrane-active agents tend to have broad activity and a low propensity for resistance development (since bacteria cannot easily alter their entire membrane composition without fitness costs).

A standout example is glabrol, a prenylated isoflavan isolated from *Glycyrrhiza glabra*. Glabrol has demonstrated rapid bactericidal effects against MRSA at low micromolar levels, attributable to membrane permeabilization and dissipation of the proton motive force (Kalli et al., 2021; Kleintjes, 2021). In time-kill assays, glabrol at 8 μg/mL completely eradicated MRSA cells within 3 h–a speed comparable to daptomycin, and notably faster than vancomycin (Sivadas et al., 2023; Tan et al., 2025). This rapid kill correlates with glabrol’s ability to insert into the bacterial lipid bilayer. It causes immediate leakage of vital ions and molecules, essentially “popping” the bacterial cell like a pin popping a balloon. Mechanistically, glabrol appears to target the anionic phospholipids that are abundant in Gram-positive membranes: the compound’s activity was strongly antagonized by exogenous phosphatidylglycerol (a major *Staphylococcus* membrane lipid), implying that glabrol binds to such lipids (Liu et al., 2025) as described in Table 2. Molecular docking studies indeed show glabrol forming hydrogen bonds with *Staphylococcus* membrane phosphatidylglycerol and cardiolipin (Kumar and Engle, 2023). By sequestering or inserting into these lipids, glabrol perturbs membrane structure and quickly dissipates the transmembrane ionic gradients, leading to bioenergetic collapse and cell death (Nicolson and Ash, 2014). The significance of this mechanism is twofold: (1) It is difficult for bacteria to develop resistance, as evidenced by glabrol’s low resistance development *in vitro* (MRSA did not readily produce glabrol-resistant mutants in serial passage tests) (Rudrapal et al., 2023). (2) It provides a means to kill dormant or non-dividing bacteria, which often survive antibiotic treatment–membrane disruptors do not rely on active cell division as β-lactams or quinolones do.

**TABLE 2:** Membrane disruption and structural damage to pathogens by licorice-derived polyphenols and related compounds.

Compound/Extract	Target pathogen(s)	Observed effects	Proposed mechanism	References
Glabridin (from Glycyrrhiza glabra)	*Staphylococcus aureus, Escherichia coli*	Leakage of intracellular contents, membrane depolarization	Interaction with lipid bilayer causing permeability changes	Yang et al. (2024)
Liquiritigenin	*Candida albicans*	Cell wall thinning, cytoplasmic disorganization	Disruption of fungal cell wall and plasma membrane integrity	Zhang et al. (2023)
Licorice total flavonoids	*Listeria monocytogenes, Bacillus subtilis*	Morphological deformation, membrane rupture	Direct insertion into phospholipid membrane	Liao et al. (2024)
Isoliquiritigenin	*Pseudomonas aeruginosa*	Leakage of nucleotides and proteins, structural collapse	Destabilization of membrane-bound enzymes and lipids	Qu et al. (2023)
Glycyrrhizic acid	*Helicobacter pylori*	Membrane blebbing and cell lysis	Alteration of lipid bilayer and increased permeability	Khan et al. (2023b)

Other licorice polyphenols share similar membrane-active properties. Licochalcone A, beyond any efflux effects, is known to perturb bacterial cell membranes and collapse the membrane potential in certain bacteria. As early as 2002, licochalcone A was reported to have potent activity against *Bacillus* spp. spores and vegetative cells by compromising their membrane integrity (Gonçalves et al., 2023). More recent work confirms that licochalcone A and its analogs (e.g., licochalcone C, licochalcone E) cause membrane depolarization in *S. aureus*, contributing to their bactericidal action (Dhaliwal et al., 2022). These chalcones are planar, lipophilic molecules that can partition into lipid bilayers–effectively acting like “wedges” that destabilize membrane packing. *Glycyrrhiza inflata* (Chinese licorice) in particular produces licochalcones A–E, which collectively showed MICs of 10–20 μg/mL against MRSA and VRE (van Dinteren et al., 2022). Their hydrophobic prenyl groups likely facilitate insertion into the largely hydrophobic membrane core, while their hydrophilic chalcone backbone can interfere with surface charge distribution. The outcome is increased permeability of the bacterial envelope, rendering the cells vulnerable.

Glycyrrhizin, though structurally very different (a glycosylated triterpene rather than a polyphenol), also exerts membrane-level effects. As a saponin-like molecule, glycyrrhizin can intercalate into lipid bilayers and form pores at sufficient concentrations–akin to soap or bile acids. It has been shown to alter membrane permeability in Gram-negatives, sensitizing them to antibiotics (Cama et al., 2019). For example, GLY treatment increased uptake of hydrophobic antibiotics in *Klebsiella pneumoniae* and *Helicobacter pylori* by disrupting outer membrane integrity (Pan et al., 2023). Furthermore, GLY’s hydrolysis product 18β-glycyrrhetinic acid is known to be membrane-active against fungi and bacteria; one study noted it could inhibit *Candida albicans* at ∼6 μg/mL, likely by binding ergosterol or membrane sterols and causing pore formation (Behbehani et al., 2023; Zhong et al., 2025). Thus, even the non-polyphenolic companion phytochemicals in licorice contribute to membrane disruption of pathogens.

It is worth highlighting that membrane-disrupting polyphenols not only kill bacteria outright, but also synergize with conventional antibiotics (De Rossi et al., 2025; Hou et al., 2024). By increasing membrane permeability, they facilitate greater antibiotic penetration. For instance, when bacterial membranes are partly compromised by licochalcone or glabrol, even efflux-prone antibiotics may enter in higher amounts and evade pump action. This synergy has been observed as a reduction in antibiotic MIC when combined with licorice extracts (Park et al., 2022). In one report, sublethal glycyrrhizic acid significantly decreased the gentamicin resistance of VRE: gentamicin (normally ineffective due to poor uptake) gained bactericidal efficacy when combined with glycyrrhizin, presumably because glycyrrhizin “primed” the bacterial membrane for drug entry (Merarchi et al., 2021). Membrane perturbation can thus be seen as a double-edged sword against MDR pathogens–it is directly lethal and it weakens the bacteria’s defensive barriers against other drugs. Given this powerful mechanism, it is unsurprising that licorice polyphenols like glabrol have been proposed as “disinfectant candidates” for surfaces and wounds (Tong et al., 2023). Indeed, glabrol was shown to rapidly eradicate MRSA on inert surfaces (simulated food preparation areas) in disinfection assays (Wang et al., 2024). Such uses could mitigate infection risk in clinical or community settings by physically eliminating pathogens where antibiotics cannot be applied.

The membrane-targeting activity of licorice polyphenols addresses one of the toughest challenges in antibiotic development: how to kill resilient bacteria without giving them an easy evolutionary escape route. Bacteria may alter a pump or enzyme to resist a conventional drug, but if their membranes are torn asunder, resistance is futile. Licorice polyphenols wield this mechanism adeptly. The key questions that remain are whether these compounds can reach the site of infection at active concentrations and whether they spare host cell membranes (specificity). Fortunately, preliminary safety evaluations are encouraging–e.g., glabrol, licochalcone A, C, and E showed minimal hemolysis or cytolysis to mammalian cells up to ∼30–50 μg/ML (Wu et al., 2019), likely because mammalian membranes contain cholesterol and zwitterionic phospholipids (like phosphatidylcholine) that render them less susceptible to these agents. This selective toxicity–disrupting bacterial but not erythrocyte membranes–is a promising feature that underscores the therapeutic potential of membrane-active licorice phytochemicals.

**FIGURE 3:**
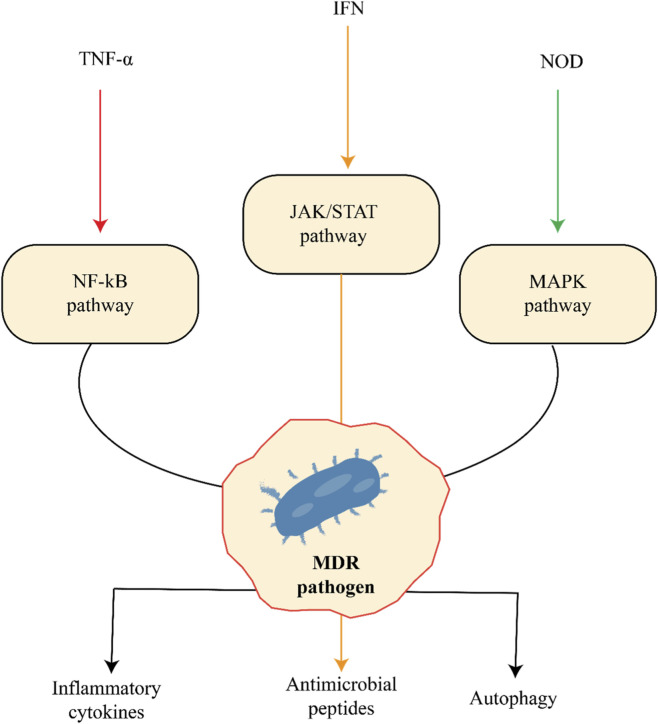
Signaling pathways involved in host defense against multidrug-resistant (MDR) pathogens.

MDR pathogens trigger multiple hosts signaling cascades, including the NF-κB pathway activated by TNF-α, the JAK/STAT pathway induced by interferons (IFN), and the MAPK pathway mediated by NOD receptors. These pathways lead to the production of inflammatory cytokines, antimicrobial peptides, and activation of autophagy, collectively contributing to the host immune response against MDR infections as show in Figure 3.

## Inhibition of biofilms and quorum-sensing

Biofilm formation is a quintessential survival strategy for bacteria under stress, contributing heavily to chronic infections and antibiotic resistance. Bacteria in biofilms can endure antibiotic concentrations tens to hundreds of times higher than planktonic cells due to the protective matrix and altered metabolic state. Therefore, anti-biofilm activity is a sought-after trait in any new antimicrobial agent aimed at MDR pathogens. Remarkably, licorice polyphenols have demonstrated significant abilities to prevent or disrupt biofilms across multiple species.

A prime example is glabridin, an isoflavane abundant in *G. glabra*. Glabridin does not just kill bacteria; at sub-inhibitory levels it can “tame” them by impeding their biofilm-forming machinery. In MRSA clinical isolates, glabridin at micromolar concentrations averted biofilm formation almost entirely (Ma et al., 2025). Crystal violet staining and electron microscopy revealed that glabridin-treated MRSA failed to develop the typical multilayered cell clusters and adhesive exopolymer matrix on surfaces (Chen et al., 2025). Instead, only sparse individual cells were observed, indicating inhibition of initial attachment and aggregation. Proteomic analysis (LC–MS of the biofilm matrix) shed light on the mechanism: glabridin significantly downregulated several surface adhesion proteins and virulence factors in MRSA (Dogheim et al., 2025). Notably, fibronectin-binding proteins (FnbA, FnbB), clumping factors, and other adhesins required for tethering bacteria to surfaces were suppressed. Meanwhile, certain “moonlighting” proteins with anti-biofilm roles (e.g., elongation factor Tu, chaperones) were inversely increased (Costantini, 2021). This suggests glabridin reprograms the surfaceome of MRSA, tipping the balance away from a biofilm-permissive state. By modulating gene expression or protein abundance related to adhesion and matrix production, glabridin essentially disarms the bacteria’s ability to form resilient communities. This is a critical function, as biofilm-associated MRSA infections (wounds, catheters, etc.) are notoriously recalcitrant–glabridin’s action could restore susceptibility in such contexts by forcing bacteria back into a planktonic, drug-sensitive lifestyle.

Other licorice-derived compounds also exhibit anti-biofilm properties, often in different bacterial species. Licorisoflavan A, 1-methoxyficifolinol, and 6,8-diprenylgenistein (polyphenols isolated from *Glycyrrhiza uralensis*) were shown to effectively prevent *Streptococcus mutans* biofilm formation on teeth-mimetic surfaces (Rudin et al., 2023). *Streptococcus mutans* is a primary agent of dental plaque (a classic biofilm); these licorice compounds interfered with its sucrose-dependent biofilm synthesis, likely by inhibiting glucosyltransferase enzymes or quorum-sensing signals needed for matrix production (Kameswaran and Ramesh, 2023). Likewise, isoliquiritigenin, a chalcone from licorice, has been reported in some studies to inhibit biofilms of *Pseudomonas aeruginosa* by quenching quorum-sensing signals (though data are preliminary). Even glycyrrhizin comes into play: while glycyrrhizin’s direct antibacterial effect is modest, it appears to inhibit biofilm maturation. One study found glycyrrhizic acid could significantly reduce *P. aeruginosa* biofilm biomass and *Staphylococcus* biofilm density when used as a co-treatment, possibly due to its anti-inflammatory and matrix-degrading synergy (licorice is known to decrease production of reactive oxygen that often promotes biofilm robustness, and it can chelate metal ions bacteria need for matrix enzymes) (Chen et al., 2024; Xiaoyan and Bin, 2023).

**FIGURE 4:**
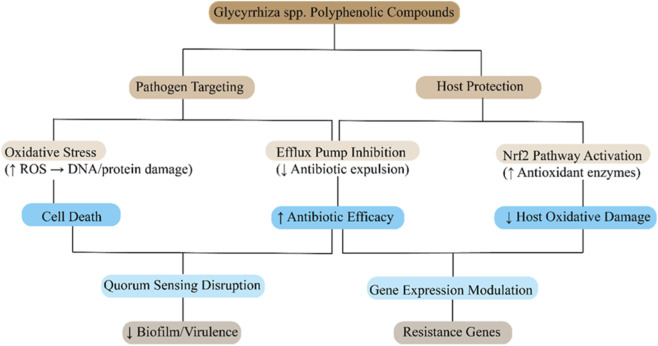
Proposed mechanistic pathways of *Glycyrrhiza* spp. polyphenolic compounds in combating antimicrobial resistance (AMR). Licorice-derived polyphenols exert dual actions through pathogen targeting (via oxidative stress induction, efflux pump inhibition, and quorum sensing disruption) and host protection (via Nrf2 pathway activation and gene expression modulation). These actions collectively enhance antibiotic efficacy, reduce virulence, limit resistance gene expression, and mitigate host oxidative damage.

Beyond direct anti-biofilm effects, licorice polyphenols may also target quorum-sensing (QS) – the bacterial cell-to-cell communication that often regulates virulence and biofilm formation as shown in Figure 4. *Glycyrrhiza* extracts have been noted in traditional use to reduce bacterial virulence in infections (e.g., licorice in respiratory infections). Modern research suggests compounds like glabridin and licochalcone may act as QS modulators. For instance, in *S. aureus*, agr-mediated QS controls the expression of surface adhesins and toxins. The observed suppression of adhesins by glabridin (Lin et al., 2023) hints that it might be interfering with the agr system or other global regulators (e.g., SarA), though this remains to be elucidated. In Gram-negatives, licorice flavonoids could potentially mimic or sequester acyl-homoserine lactone signals, thus attenuating virulence factor expression. While less explored than efflux or membrane effects, the ability of licorice polyphenols to silence bacterial chatter and thereby reduce virulence is an attractive supplementary mechanism. It means these compounds might not need to outright kill pathogens in order to be clinically useful–by preventing biofilm formation and suppressing toxin production, they can render infections more benign and easier to clear with immune mechanisms or conventional antibiotics (Hotinger et al., 2021).

In summary, licorice polyphenols contribute to anti-MDR activity not only by killing bacteria but by undermining bacterial community behavior and resilience. Biofilm inhibition is particularly noteworthy for risk mitigation: disrupting biofilms can prevent chronic infection establishment (e.g., on indwelling medical devices) and can help break down barriers in established infections so that antibiotics and immune cells can penetrate. The evidence thus far, from MRSA to oral streptococci, paints licorice compounds as potent anti-biofilm agents at sub-lethal doses. This multi-pronged approach–hit the bugs’ pumps, membranes, *and* biofilms–is what sets polyphenols apart from many single-target antibiotics, and it underscores the unique value of *Glycyrrhiza* phytochemicals in the fight against superbugs.

## Synergy with conventional antibiotics and therapeutic potential

**FIGURE 5:**
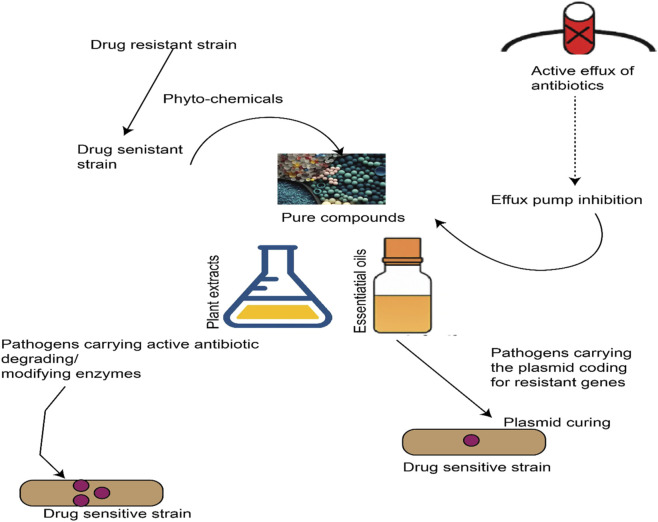
Schematic representation of antibiotic interaction with bacterial cells. Antibiotic compounds target bacterial structures and functions, leading to growth inhibition and cell death. The illustration highlights drug penetration, disruption of cellular integrity, and suppression of bacterial proliferation, representing the general mechanisms of antibacterial action that underlie therapeutic control of pathogenic infections.

A recurring theme in the above mechanisms is synergy: licorice polyphenols often work best not in isolation but in concert with standard antibiotics. This aligns with an emerging paradigm in AMR management–using combination therapy to outflank resistance. Licorice compounds can be viewed as resistance-modifying agents or adjuvants that enhance the efficacy of antibiotics that pathogens have learned to withstand as shown in Figure 5. There are several concrete examples of this synergy:

- Efflux Pump Synergy: Glabrene dramatically potentiated fluoroquinolone activity by inhibiting NorA efflux in *S. aureus*, cutting the required antibiotic dose by up to eightfold (Ika Irianti et al., 2023). In practical terms, this could revive the use of fluoroquinolones against MRSA when combined with a licorice isoflavonoid–a combination that would have been thought pointless (since MRSA pumps the drug out) now becomes effective. This principle can extend to other efflux-mediated resistance; many Gram-negatives rely on RND efflux pumps for intrinsic resistance, and licorice phytochemicals that impair those pumps could similarly lower the MICs of carbapenems or tetracyclines against those organisms (Rai, 2025).
- Membrane Synergy: Membrane-disrupting polyphenols like licochalcone A or glabrol can render bacteria more permeable to hydrophilic antibiotics or large molecules that normally struggle to penetrate. A striking case was glycyrrhizin’s effect on gentamicin-resistant bacteria: in glycyrrhizin-treated cultures of vancomycin-resistant *Enterococcus*, the MIC of gentamicin (an aminoglycoside) was dramatically reduced, indicating synergy (Chen et al., 2024). Gentamicin resistance in Enterococci often stems from poor drug uptake and modifying enzymes; by permeabilizing membranes or modulating permeability, glycyrrhizin allowed more gentamicin into the cell and possibly overwhelmed any low-level resistance mechanism described in Table 3 (Patterson and Zervos, 1990). The outcome was that previously resistant Enterococci became susceptible to gentamicin when combined with glycyrrhizin (Li et al., 2022). This finding is encouraging for the concept of pairing traditional antibiotics with natural compounds to restore efficacy. Similar synergistic effects have been observed with licorice extracts plus β-lactams against MRSA, and with licorice polyphenols plus azoles against drug-resistant fungi (attributed to membrane effects that enhance drug uptake).
- Biofilm/Immunomodulatory Synergy: Licorice compounds also synergize by improving host conditions for antibiotics to work. For example, licorice extract in a burn wound model (mice) significantly reduced *P. aeruginosa* infection severity not by directly killing the bacteria, but by boosting the skin’s production of antimicrobial peptides (defensins) and mitigating the immunosuppressive effects of burn injury (Chen et al., 2024). Glycyrrhizin was the active component in that study–it helped the damaged tissue mount an innate immune response (peptides that punch holes in bacteria) and likely made the residual bacteria more exposed to any topical antibiotics or immune clearance (Chen et al., 2024). In another angle, the anti-biofilm effect of glabridin could synergize with antibiotics by breaking the biofilm barrier, as mentioned (Ma et al., 2025). A biofilm-disrupted bacterial population will be hit much harder by a given antibiotic dose than an intact biofilm population. One could envision a “two-hit” therapy: first treat with a licorice polyphenol to disperse the biofilm or weaken defenses, then administer a conventional antibiotic to kill the now-vulnerable bacteria.
- Specific Combinations: Early-stage research has tested specific licorice compounds in combination with established drugs. For instance, glabridin + nisin (a peptide antibiotic) was found to be highly synergistic against *Enterococcus faecalis* biofilms, completely eradicating cells where either agent alone only partially worked (Khan et al., 2023a; Ma et al., 2025). Here, glabridin’s membrane and biofilm inhibition combined with nisin’s pore-forming ability led to a one-two punch that neither the bacterial cell wall nor biofilm matrix could withstand. Another intriguing combination is licorice polyphenols with colistin (a last-resort polymyxin antibiotic): some Gram-negatives resistant to colistin via modified LPS were resensitized when treated with isoliquiritigenin, presumably because the chalcone interfered with the lipid modifications or added its own membrane perturbation, allowing colistin to bind and disrupt the outer membrane again (Mondal et al., 2024).

**TABLE 3:** Synergistic effects of flavonoids with conventional antibiotics and their therapeutic potential.

Flavonoid compound	Antibiotic partner	Target pathogen(s)	Observed synergistic effect	Proposed mechanism of synergy	References
Quercetin	Ciprofloxacin	*Staphylococcus aureus* (MRSA)	Enhanced bactericidal activity, reduced MIC	Disruption of efflux pumps, increased antibiotic uptake	Amin et al. (2015), Tan et al. (2025)
Epigallocatechin gallate (EGCG)	β-lactams	*Escherichia coli, Pseudomonas aeruginosa*	Restored β-lactam sensitivity	Inhibition of β-lactamase activity	Pacyga et al. (2024)
Baicalein	Tetracycline	*Staphylococcus aureus*	Increased inhibition of bacterial growth	Membrane permeabilization enhancing antibiotic penetration	Moulick and Roy (2024)
Luteolin	Vancomycin	*Enterococcus faecalis*	Potentiated antibacterial activity	Synergistic binding to cell wall synthesis targets	Lu et al. (2023)
Hesperidin	Gentamicin	*Klebsiella pneumoniae*	Decreased bacterial viability	Reactive oxygen species (ROS) generation and membrane disruption	Guo et al. (2023)
Naringenin	Chloramphenicol	*Salmonella typhi*	Significant reduction in MIC values	Interference with protein synthesis and improved antibiotic access	Khan et al. (2025)

Taken together, these findings suggest that *Glycyrrhiza* polyphenols could be deployed not necessarily as standalone antibiotics (although a few are potent enough to consider for that role), but as adjuvant therapies to existing antibiotics. Such an approach has multiple advantages: it may prevent the emergence of resistance (because bacteria now have to counter two very different agents simultaneously), it can recycle old antibiotics that had fallen out of use due to resistance, and it may allow lower doses of antibiotics to be used (reducing toxicity). This is essentially a risk mitigation strategy–using licorice phytochemicals to reduce the risk that infections will become untreatable by our antibiotic arsenal. Indeed, researchers have advocated for more studies on combining plant-derived compounds with antibiotics as a way to extend the lifespan of current drugs (Abdallah et al., 2023; Chaachouay and Candidates, 2025). Licorice, with its millennia of use and GRAS (generally regarded as safe) status in certain contexts (e.g., as a flavoring), is a particularly attractive source for such adjuvants.

However, synergy *in vitro* does not automatically translate to clinical success (Wang et al., 2025). The timing, dosing, and delivery method for combination therapy need careful optimization. It is crucial to evaluate pharmacokinetic compatibility, for instance, whether a licorice-derived flavonoid can achieve adequate concentrations in the bloodstream or infection site concurrently with the administered antibiotic, Or is a topical/local delivery more effective (such as a wound dressing impregnated with licorice extract to pair with systemic antibiotics)? These practical considerations are discussed next, as we shift to evaluating the safety, toxicity, and formulation challenges that must be addressed to bring licorice polyphenols into real-world use.

## Safety, toxicology, and pharmacokinetic considerations

Any agent intended for therapeutic use–even a natural product–must undergo rigorous evaluation for safety and proper pharmacokinetics (PK). Polyphenols and saponins from licorice have a history of human exposure through diet and herbal medicine, which provides some baseline reassurance but also highlights certain toxicity concerns at high doses (Emerald, 2024). This section examines what is known about the safety profiles of licorice polyphenols, their dose-response behavior (including hormesis), and the challenges in delivering them effectively to infection sites.

Toxicity and Side Effects: The most famous licorice constituent, glycyrrhizin (glycyrrhizic acid), is paradoxically both a therapeutic agent and a source of toxicity when overconsumed (Dang et al., 2024). Glycyrrhizin can cause mineralocorticoid-like side effects (pseudoaldosteronism) by inhibiting an enzyme (11β-HSD2) that inactivates cortisol. Excessive licorice intake is known to lead to hypertension, edema, hypokalemia, and electrolyte imbalances–essentially a syndrome of *apparent mineralocorticoid excess* (Ceccuzzi et al., 2023). These effects are well documented in clinical literature and led regulatory agencies to recommend limits on licorice consumption in foods. For context, chronic ingestion of >50 g licorice candy per day (containing glycyrrhizin) has precipitated such symptoms. In medicinal contexts, glycyrrhizin dosing is usually kept moderate (e.g., ∼40–100 mg/day intravenously for hepatitis as in the Japanese SNMC formulation) (Giangrandi et al., 2024). These doses are generally well tolerated. The relevance here is that if glycyrrhizin or licorice extracts were used systemically for antimicrobial purposes, monitoring for these metabolic side effects would be necessary, especially in patients with cardiovascular or renal vulnerabilities.

Many of the polyphenols in licorice lack glycyrrhizin’s specific endocrine effects and tend to have different toxicity considerations. Flavonoids like glabridin, licochalcone A, glabrol, etc., do not cause pseudoaldosteronism (Sabbadin et al., 2024). Their toxicity is more related to general cell or organ effects at high concentrations. Encouragingly, studies indicate that licorice polyphenols have a wide therapeutic window *in vitro*. For example, as noted earlier, glabrol and licochalcones caused negligible lysis of red blood cells up to 128 μg/mL and had IC_50_ values >25 μg/mL on mammalian cell lines (Tuli et al., 2022), whereas their MICs against MRSA were in the 2–8 μg/mL range (Bawankar et al., 2024). This selectivity (>5-fold) is quite favorable. Glabridin likewise showed no cytotoxic effect on human keratinocytes at concentrations that inhibit bacterial biofilms, and animal studies using glabridin in topical formulations reported no significant dermal irritation (Li et al., 2025). An important factor in safety is that many polyphenols exhibit antioxidant and anti-inflammatory properties, which can counterbalance any pro-oxidant or irritant effects. Glabridin, for instance, is a known antioxidant that scavenges free radicals; in a wound or infection scenario, it might reduce tissue damage from inflammation even as it attacks bacteria (Ugoeze and Odeku, 2025).

One must consider hormesis–the phenomenon where a compound has beneficial effects at lower doses but harmful effects at high doses. Polyphenols are classic hormetic agents in many biological systems (e.g., dietary polyphenols activate stress-response pathways at low doses but can be pro-oxidant cytotoxins at high doses) (Murakami, 2024). In the context of licorice polyphenols, a hormetic perspective means that we might observe stimulatory or protective effects at sub-lethal concentrations (like induction of host defense peptides or mild activation of antioxidant defenses), whereas at high concentrations these compounds become indiscriminately damaging (e.g., high glabrol could lyse not only bacteria but also start solubilizing host cell membranes). Achieving the right balance–maximizing the hormetic benefit to the host and the toxic effect to the microbe without crossing into host toxicity–will be key. Encouragingly, the effective antimicrobial concentrations of many licorice compounds are near or below their toxicity threshold in cell assays (Hoseinpoor et al., 2024). Furthermore, the anti-inflammatory activity of glycyrrhizin and certain flavonoids might allow them to mitigate their own collateral damage; for example, glycyrrhizin’s ability to inhibit HMGB1 and TNF-α release could reduce tissue inflammation during infection treatment, an added safety bonus (Chen and Di, 2024). In any case, comprehensive toxicological studies in animal models (acute and chronic dosing) are needed for each candidate compound to ensure that the therapeutic index is acceptable.

Pharmacokinetics and Bioavailability: A notorious challenge with natural polyphenols is poor bioavailability. Many are lipophilic and have low aqueous solubility, and those that are polar often get extensively metabolized or conjugated in the gut and liver (Klebe, 2025). Licorice polyphenols are no exception. Glabridin, for example, is lipophilic (logP ∼3) and not very water-soluble; oral absorption is limited and it undergoes first-pass metabolism. Studies have shown that only a small fraction of ingested glabridin reaches systemic circulation, and it is rapidly distributed into tissues (especially adipose) due to its lipophilicity. Licochalcone A is similarly hydrophobic and susceptible to metabolism (Phase I reduction of the chalcone and Phase II glucuronidation) (Liu et al., 2024). Glycyrrhizin, despite being a polar glycoside, is actually not absorbed intact; gut bacteria cleave it to glycyrrhetinic acid, which is then absorbed and circulates (Liu et al., 2024). Glycyrrhetinic acid has a longer half-life and is the moiety that causes mineralocorticoid effects. The PK of glycyrrhizin has been studied in humans (Han et al., 2024): when given IV, it has a multi-phase elimination, with glycyrrhetinic acid peaking hours after dosing due to slow formation and then clearing over a day or more (it can accumulate with daily dosing). This *prodrug* aspect might be advantageous for some infections (e.g., colonic infections, where glycyrrhizin could travel intact to the colon, then release glycyrrhetinic acid locally via microbiota–essentially doing “targeted delivery” to gut pathogens). But for systemic infections, glycyrrhizin’s PK complicates dosing; intravenous routes avoid the gut metabolism but at cost of potential toxicity.

To ensure licorice polyphenols reach their targets, formulation science is pivotal. Researchers have been exploring various delivery strategies: *nanoformulations* (nanoparticles, liposomes, micelles) to solubilize and protect the compounds, *derivatization* (prodrug approaches, or increasing water-solubility via glycosylation/PEGylation), and *localized delivery systems*. For instance, a glycyrrhizin-based hydrogel was developed for wound healing applications, which slowly releases the compound at the wound site (Mees et al., 2022). This hydrogel significantly improved healing of MRSA-infected wounds in a mouse model, indicating that a topical formulation can achieve high local concentrations without systemic exposure (Aliakbar Ahovan et al., 2022; Chegini et al., 2025). Similarly, incorporating licorice extracts into antimicrobial coatings for medical devices is under investigation–one study impregnated cotton fabric with *Glycyrrhiza* extracts and found it conferred antibacterial properties to the textile (Adeel et al., 2022). Such approaches could be directly relevant to reducing hospital-acquired infections (e.g., licorice-infused catheter coatings to prevent biofilms).

Another approach is combination formulation: co-delivering the polyphenol with an antibiotic in the same carrier. If we want glabrene to boost ciprofloxacin, a nanocarrier that encapsulates both might ensure they concentrate in the same bacterial cell. There is also interest in inhalable formulations for respiratory infections (licorice has been traditionally used for cough and lung ailments). An aerosol containing a licorice flavonoid and a low dose of antibiotic could target MRSA pneumonia or *P. aeruginosa* in cystic fibrosis lungs, for instance (Umarov et al., 2025). Inhalation bypasses some systemic PK issues and delivers the drug where needed.

In terms of metabolism (ADME), we should consider potential interactions. Polyphenols can inhibit certain cytochrome P450 enzymes or P-glycoprotein; for example, glabridin is known to inhibit CYP3A4 *in vitro*, which could raise levels of co-administered drugs metabolized by that pathway (Vieira et al., 2025). Glycyrrhetinic acid can inhibit OATP transporters in the liver. These interactions need careful examination to avoid adverse drug interactions when licorice compounds are used alongside antibiotics or other medications. On the flip side, such effects might also beneficially slow the clearance of some antibiotics, effectively boosting antibiotic levels (though this would be a double-edged sword due to toxicity risks).

Hormetic Dose Optimization: To tie together safety and PK–the concept of hormesis suggests that careful dosing could maximize benefit/minimize harm. Perhaps sub-antimicrobial doses of licorice polyphenols could be given chronically to prime host defenses and weaken bacteria, without reaching levels that cause toxicity (Chen et al., 2024). Then, antibiotics at standard doses finish the job. This two-tier strategy might avoid the need to push polyphenol doses into a range that risks toxicity. There is some evidence that chronic low-dose glycyrrhizin can have immunomodulatory benefits (as seen in reduction of inflammatory cytokines and improved outcomes in chronic hepatitis patients (Richard, 2021). If similar low doses can affect bacterial virulence (e.g., inhibit biofilms or efflux) over time, they could serve as a prophylactic adjunct in patients at high risk for MDR infections (such as those with implanted devices or recurrent UTIs). Of course, such prophylaxis would have to be weighed against any long-term toxicity; so far, low-dose licorice in diets (like licorice tea) is mostly benign, but cumulative effects need monitoring.

In summary, while licorice polyphenols show great promise in the Petri dish and in small-animal studies, translational hurdles remain. Ensuring these compounds are safe at effective doses is paramount. The known side effects of glycyrrhizin impose caution, but with rational design (using the right compounds for the right route), it is feasible to mitigate those risks. Modern formulation techniques offer solutions to bioavailability challenges, potentially turning these ancient herbal molecules into cutting-edge therapeutics or preventive materials. Next, we consider the broader translational and clinical gaps–what is needed to move from promising laboratory data to approved clinical interventions.

## Polyphenols against drug-resistant infections

### Resveratrol

In mouse/rat infection models, resveratrol has shown marked anti-Staphylococcus efficacy *in vivo*. Topical resveratrol accelerated healing of S. aureus–infected skin wounds: in one murine dermal wound model (2 × 2 cm abrasion infected with 10^9 CFU *S. aureus*), resveratrol treatment increased wound closure to ∼76% by day 14 *versus* ∼60–67% in controls (Shevelev et al., 2020). Histology showed resveratrol reduced neutrophilic inflammation and shifted immunity toward a Th2/healing profile (Shevelev et al., 2020). In a mouse air-pouch infection with *S. aureus*, photodynamic activation of resveratrol (blue light excitation) dramatically boosted innate clearance: treated mice had higher TNF-α/IL-17A, lower bacterial load and inflammation after 24 h (Dos Santos et al., 2019). These data suggest resveratrol (especially in bioactivated or formulated form) can directly stimulate host defense and inhibit *S. aureus in vivo*. Resveratrol is generally well-tolerated, though its low oral bioavailability is noted; efforts focus on advanced delivery systems to sustain its activity (Cui et al., 2024).

### Curcumin

Curcumin has demonstrated efficacy against MRSA and other bacteria *in vivo*, often in combination with antibiotics. In a murine orthopedic implant model of MRSA osteomyelitis (USA300 strain), daily i. p. curcumin (20 mg/kg) together with gentamicin (20 mg/kg) significantly improved outcomes: treated mice showed markedly reduced peri-implant biofilm burden, preserved trabecular bone architecture and diminished inflammatory osteolysis compared to gentamicin alone (Chen et al., 2022). Likewise, in a mouse burn wound infection model, topical curcumin substantially reduced wound size and bacterial load (Hussain et al., 2022). In fact, by day 16 post-infection curcumin dressings led to significantly faster closure of S. aureus–infected burns than controls (p < 0.05 (Hussain et al., 2022)). These *in vivo* studies (using doses on the order of 10–20 mg/kg or equivalent topical doses) confirm curcumin’s broad anti-Staphylococcal and anti-Escherichia effects and its ability to enhance antibiotic therapy. Curcumin’s mechanisms include direct toxin inhibition (e.g., binding α-hemolysin) and quorum-sensing disruption (Hussain et al., 2022; Wang et al., 2016). Safety profiles in animal and human studies are favorable, but curcumin’s poor bioavailability has spurred the use of nanoparticles or adjuvants in trials (e.g., liposomal or nanoparticle curcumin) to achieve effective tissue levels.

### Quercetin

A Chinese study (2022) showed that quercetin greatly reduced MRSA virulence *in vivo* by targeting the bacterial ClpP protease. In a mouse model of lethal MRSA pneumonia (likely USA300 strain), treatment with quercetin (∼64 μg/mL *in vitro*, dosing *in vivo* not specified) dramatically improved survival. Quercetin bound ClpP and inhibited its activity, down-regulating MRSA toxin genes (hla, agr, psm-α etc.) and attenuating hemolysis (Jing et al., 2022). The net effect was “effective protection” of infected mice: quercetin-treated animals survived otherwise fatal MRSA lung infection. This study highlights quercetin’s antivirulence action and suggests a new therapeutic strategy against resistant *S. aureus*. Other flavonoids with similar modes (e.g., myricetin, apigenin) have been proposed but *in vivo* data are emerging primarily for quercetin.

### Catechins

Epigallocatechin-3-gallate (EGCG), the major green-tea catechin, has shown potent *in vivo* activity against Gram-negative pneumonia. In one study, mice were given oral EGCG daily (20, 40 or 80 mg/kg) for 3 days, then infected intratracheally with *Pseudomonas aeruginosa* PAO1 (∼2.5 × 10^8 CFU). EGCG markedly reduced lung edema and pathology: bacterial CFUs in lung tissue fell significantly and pro-inflammatory cytokines (TNF-α, IL-17) were suppressed. Importantly, EGCG dramatically improved host survival (Tang et al., 2022). At 80 mg/kg, survival approached that of ciprofloxacin-treated controls, but EGCG also uniquely boosted IL-10 and IL-4 (anti-inflammatory cytokines), indicating immunomodulation. Molecular analyses showed EGCG downregulated *P. aeruginosa* quorum-sensing and virulence genes (lasI/R, rhlI/R, pqsA/R, phz operon, lasA/B, rhlA/C) *in vivo* (Tian et al., 2025). These findings confirm that dietary catechins can both reduce Gram-negative bacterial burden and dampen damaging inflammation in infection. Similar EGCG effects have been reported in models of *Salmonella* enteritis (reduced gut colonization and inflammation) and *Escherichia coli* infections, though dosing and routes vary.

### Other polyphenolic agents

A few recent studies on related compounds are noteworthy. In a mouse wound-infection model, a combination of isobavachalcone (a prenylated chalcone) and curcumin with gentamicin eradicated MRSA biofilm on implants and protected bone (Chen et al., 2022). This suggests that polyphenol cocktails can synergize with antibiotics *in vivo*. Another example is naringenin or baicalein (not detailed here) which have shown immunoprotective effects in sepsis models. To date, clinical trials of polyphenols specifically for drug-resistant infections are scarce. However, the *in vivo* preclinical data demonstrate clear therapeutic potential: these compounds are generally non-toxic at tested doses, reduce pathogen load in animal models (often at 10–100 mg/kg), and modulate host inflammation. Their mechanisms (antitoxin binding, QS-inhibition, efflux pump inhibition, immune activation) have been validated *in vivo* and imply that polyphenols could serve as adjuvants or standalone therapies against multidrug-resistant *S. aureus*, *P. aeruginosa*, *E. coli* and other pathogens (Chen et al., 2022; Dos Santos et al., 2019; Tang et al., 2022).

## In silico evidence for polyphenol-pathogen interactions

In silico approaches, including molecular docking and molecular dynamics (MD) simulations, have provided valuable predictive insights into the interactions of licorice polyphenols with bacterial targets implicated in AMR (Rudrapal et al., 2023). These computational studies complement experimental data by elucidating binding modes, affinities, and stability against efflux pumps, biofilm matrices, and membrane proteins in MDR/ESKAPE pathogens (Giorgini, 2024).

For instance, isoliquiritigenin and glabridin have demonstrated favorable docking scores (binding energies often < -7 kcal/mol) and stable MD trajectories (low RMSD/RMSF fluctuations) when interacting with efflux pump proteins (e.g., NorA in *Staphylococcus aureus*) and biofilm-associated components, suggesting mechanisms of efflux inhibition and biofilm disruption (Madkhali et al., 2026). Similarly, glycyrrhizin and liquiritigenin show high-affinity binding to β-lactamase-like enzymes and membrane porins, predicting reduced enzymatic degradation of antibiotics and enhanced membrane permeability in Gram-negative MDR strains (Pages et al., 2008).

Recent studies further highlight that chalcones from Glycyrrhiza (e.g., isoliquiritigenin) exhibit synergistic potential by modulating resistance pathways, with computational predictions aligning with observed restoration of antibiotic susceptibility in resistant strains (Al-Azzawi et al., 2024). These *in silico* results underscore the multi-target potential of licorice polyphenols, supporting their role in overcoming efflux, biofilm, and target modification mechanisms (Wu et al., 2025).

## Gaps in translational research and clinical validation

Despite the compelling *in vitro* and preclinical data on *Glycyrrhiza* polyphenols, there remains a pronounced gulf between laboratory research and clinical application. No licorice-derived polyphenol is currently an approved antimicrobial drug or adjuvant in mainstream medicine. Bridging this gap requires addressing several issues:

- Limited Clinical Trials: To date, clinical studies of licorice have focused on its antiviral (e.g., hepatitis, HIV) and gastroprotective effects, not on treating bacterial infections. For instance, glycyrrhizin in the form of Stronger Neo-Minophagen C has been trialed in hepatitis patients with success (Mou et al., 2024), but there is a lack of clinical trials evaluating licorice extracts or isolated polyphenols in bacterial infection scenarios. One exception is the use of licorice in traditional cough remedies and *Helicobacter pylori* management, but rigorous trials are scarce. This means we lack real-world data on efficacy against MDR pathogens in humans. Translational research should prioritize moving the most promising candidates (like glabrol or glabridin) into Phase I safety trials and Phase II efficacy trials for specific indications (e.g., diabetic foot infections with MRSA, or VRE colonization in patients where an oral non-absorbable agent like glycyrrhizin might clear gut carriage).
- Pharmaceutical Development Challenges: Isolating or synthesizing these polyphenols at scale and at high purity is non-trivial. Licorice root extracts contain dozens of constituents; making a consistent drug product from them requires either standardized extracts (with quality control on key active concentrations) or total synthesis/semisynthesis of individual compounds. Total synthesis of complex polyphenols (especially prenylated, stereochemically rich ones like glabrol) can be challenging and costly. However, some simpler analogs might be obtainable. Alternatively, plant cell culture or microbial fermentation could be explored to produce specific licorice compounds. From a regulatory perspective, defining a polyphenol drug’s identity, purity, and stability will be essential. The distinct structure of these molecules (e.g., glabridin is an isoflavan with two chiral centers) means stability testing under various conditions (light, heat, pH) to ensure shelf life. Many polyphenols are prone to oxidation or polymerization over time, which could complicate formulation–thus research on stabilizers or antioxidants to accompany them might be needed.
- Target Validation *in Vivo*: It is one thing to show a compound inhibits an efflux pump in a bacterial culture; it is another to prove it does so in an infected human body. The *in vivo* environment has many additional factors–serum proteins that bind polyphenols (possibly reducing free active concentration), immune system interactions, and the complexity of infection sites (abscess pH, anaerobic conditions, etc.). We need more animal models to test licorice polyphenols in realistic infection scenarios: for example, does combining glabrene with levofloxacin cure MRSA mouse infections better than levofloxacin alone? Does topical glabridin reduce MRSA burden in a pigskin wound model? Some progress has been made (glabrol was tested in a Galleria mellonella insect model and showed improved survival of larvae with MRSA infection (Ménard et al., 2021), but mammalian models will provide stronger evidence. These studies should also monitor not just efficacy but any signs of toxicity or interference with host healing.
- Spectrum and Resistance Development: More investigation is needed into the spectrum of activity of each compound and the risk of bacteria developing resistance to them. For example, many licorice polyphenols are more active against Gram-positive bacteria than Gram-negatives (likely due to outer membrane exclusion) (Yang et al., 2022). This is not a deal-breaker–Gram-positives (MRSA, VRE, *C. difficile*) are themselves critical threats, and Gram-negatives might still be addressed via adjuvant effects–but it is important to delineate where each compound is best applied. Could *Pseudomonas* easily pump out licochalcone A, or modify its membrane to resist glabrol? So far, glabrol’s rapid kill seems to forestall typical resistance, but if used sublethally, bacteria might adapt (perhaps by altering membrane charge or lipid composition). Continuous culture experiments could check for emergent resistance phenotypes. Also, combination therapy might reduce the risk of resistance to the polyphenols themselves (since the antibiotic partner kills most bacteria, leaving few to adapt to the polyphenol), but this assumption needs validation (Vladu et al., 2022).
- Interactions with Human Microbiome: When administering any antimicrobial, we must consider collateral effects on the beneficial microbiota. Glycyrrhizin taken orally, for instance, will encounter gut flora and may inhibit some commensals or, conversely, be metabolized by them (as indeed it is). How do licorice polyphenols affect gut microbiome composition? Interestingly, some studies suggest licorice extracts can *foster beneficial bacteria*–for example, by inhibiting pathogens like *H. pylori* (Addissouky et al., 2023) or *Clostridioides difficile* more than commensals, or by acting as a substrate for certain gut microbes that hydrolyze glycosides. This area is under-explored but is relevant if considering systemic or oral use of licorice compounds. The ideal scenario is that they selectively target pathogens (especially those often in dysbiosis or infection states) and spare or even promote healthy flora. Nonetheless, thorough profiling of microbiome changes in any eventual clinical trials would be wise.
- Regulatory and Perceptual Hurdles: Using an herbal-derived substance in a high-impact medical context faces both regulatory scrutiny and some skepticism. Regulatory agencies will treat a purified licorice polyphenol as a drug, requiring full toxicological and clinical evaluation. Alternatively, a *standardized licorice extract* might be considered a botanical drug (like some green tea extract ointments approved for warts). The pathway needs to be clarified early. Moreover, clinicians may need convincing: the concept of “licorice for superbugs” could be met with doubt if not backed by robust data, given the historical reliance on synthetic antibiotics. Thus, a part of translational effort is also educational and reframing–presenting licorice polyphenols not as folk remedies but as evidence-based, mechanism-of-action-driven agents that fit into modern antimicrobial strategies (perhaps analogized to how quinine from cinchona led to quinolone drugs, etc.).

In highlighting these gaps, we also illuminate opportunities. Each gap directs us to a necessary future research thrust: conduct pilot clinical trials in a focused indication (e.g., a topical licorice polyphenol for MRSA nasal decolonization), develop advanced formulations to improve delivery, perform combination studies in animal infection models, and investigate safety margins deeply. By addressing these systematically, the field can advance licorice polyphenols from bench curiosity to clinical reality in the fight against MDR pathogens.

## Conclusion and future outlook

Multidrug-resistant pathogens represent a global hazard on par with climate change and pandemics, demanding innovative countermeasures. Polyphenolic compounds in Glycyrrhiza spp. offer a multi-faceted solution: they are naturally equipped to undermine bacteria’s survival strategies–from efflux pumps to biofilms–while also directly killing the invaders and working synergistically with our existing antibiotics. This review has critically examined how licorice-derived flavonoids, chalcones, coumarins, and glycosides can tilt the scales back in our favor against “superbugs.” The evidence, drawn largely from primary research, paints a compelling picture: licorice polyphenols like glabrol, glabridin, licochalcone A, and glycyrrhizin can either punch holes in bacterial defenses or patch the holes in our antibiotic arsenal–and often do both.

To distinguish this work from a conventional narrative review, the presented evidence was interpreted within a framework of risk mitigation and hazard management. Antimicrobial resistance (AMR) represents a major biological hazard, whereas licorice-derived polyphenols may function as potential mitigating agents. These compounds illustrate how natural chemical diversity can be harnessed to address a largely anthropogenic crisis—namely, antibiotic resistance resulting from extensive and often inappropriate antimicrobial usage. Importantly, the role of these phytochemicals extends beyond that of classical antibiotics. Instead, they function as adjuvants, resistance modulators, and synergistic enhancers, thereby offering new conceptual directions for combination-based therapeutic strategies. A mechanism-oriented evaluation—encompassing efflux pump inhibition, membrane destabilization, biofilm interference, and synergistic interactions—demonstrates that licorice polyphenols target key bacterial physiological pathways that are traditionally difficult to modulate without inducing host toxicity.

In summary, polyphenolic constituents of Glycyrrhiza represent a biologically ancient resource being strategically repurposed to confront the contemporary challenge of antimicrobial resistance. Their capacity to potentiate existing antibiotics, attenuate microbial defense systems, and potentially influence host immune responses is well aligned with the multifaceted approaches required to combat multidrug-resistant (MDR) pathogens. Although substantial translational research is still necessary to advance these natural compounds toward clinical application, current findings indicate considerable promise. Future developments may enable licorice-derived molecules to progress from experimental investigations to clinical or applied settings, either as components of combination therapies or as innovative antimicrobial materials. Such advancements would not only substantiate long-standing ethnopharmacological knowledge surrounding licorice but also contribute meaningful new strategies against the escalating global threat of antibiotic resistance. In the context of one of the most critical health challenges of the 21st century, Glycyrrhiza polyphenols may emerge as both scientifically credible and therapeutically valuable allies.
